# Rationale, design and baseline characteristics of the TRANSCEND-CKD trial of retatrutide in patients with chronic kidney disease

**DOI:** 10.1093/ndt/gfaf230

**Published:** 2025-10-29

**Authors:** Hiddo J L Heerspink, Daniël H van Raalte, Petter Bjornstad, Mathijs C Bunck, Peikun Wu, Ilke Tunali, Zvonko Milicevic, Lisette Koeneman

**Affiliations:** Department of Clinical Pharmacy and Pharmacology, University Medical Center Groningen, University of Groningen, Groningen, The Netherlands; Amsterdam University Medical Center, Amsterdam, The Netherlands; University of Washington School of Medicine, Seattle, WA, USA; Eli Lilly and Company, Indianapolis, IN, USA; R&G US, Somerset, NJ, USA; Eli Lilly and Company, Indianapolis, IN, USA; Eli Lilly and Company, Indianapolis, IN, USA; Eli Lilly and Company, Indianapolis, IN, USA

**Keywords:** chronic kidney disease, glucagon, incretins, iohexol-measured GFR, retatrutide

## Abstract

**Background and hypothesis:**

Retatrutide is an agonist of the glucose-dependent insulinotropic polypeptide, glucagon-like peptide-1 and glucagon receptors that reduced weight and hemoglobin A_1c_ (HbA_1c_) in individuals with obesity and type 2 diabetes (T2D). Retatrutide may also address key chronic kidney disease (CKD)-related pathophysiological pathways. Mechanism-of-action studies are needed to understand its effects on kidney function.

**Methods:**

TRANSCEND-CKD is a double-blind, placebo-controlled, Phase 2b mechanistic study evaluating the efficacy of retatrutide in adults with overweight/obesity and CKD [estimated glomerular filtration rate (eGFR) 25–75 mL/min/1.73 m^2^], with and without T2D. Participants were randomized 1:1 to once-weekly retatrutide maximum tolerated dose up to 12 mg or matched placebo. The primary objective is to evaluate the effect of retatrutide versus placebo on change in measured glomerular filtration rate (mGFR) by iohexol clearance from baseline to Week 24. Additional objectives include changes in magnetic resonance imaging–assessed kidney hemodynamic and volumetric measurements, including perirenal and renal sinus fat.

**Results:**

Of 367 participants screened, 146 were randomized to study interventions. The mean age was 65.1 years [standard deviation (SD) 10.6], 45.2% were female and 69.9% were White. The mean weight was 101.1 kg (SD 20.6) and body mass index 35.7 kg/m^2^ (SD 6.1). Participants with T2D (37.7%) had a mean HbA_1c_ of 7.1% (SD 1.1%), while HbA_1c_ was 5.7% (SD 0.3%) in participants without T2D. The mean mGFR was 49.3 mL/min/1.73 m^2^ (SD 19.0), cystatin C–based eGFR was 49.6 mL/min/1.73 m^2^ (SD 13.2) and creatinine-based eGFR was 64.2 mL/min/1.73 m^2^ (SD 17.8). The median baseline urine albumin-to-creatinine ratio was 14.0 mg/g (interquartile range 6.0–69.0). A total of 21.8% of participants were treated with sodium-glucose cotransporter-2 inhibitors at baseline.

**Conclusions:**

TRANSCEND-CKD is designed to provide mechanistic insights on the effects of retatrutide on kidney function and structure, and to inform clinical findings in the ongoing cardio-kidney outcome trial TRIUMPH-Outcomes (NCT06383390).

**Clinical Trial Registration Number:**

NCT05936151

## Graphical Abstract

**Graphical Abstract:**
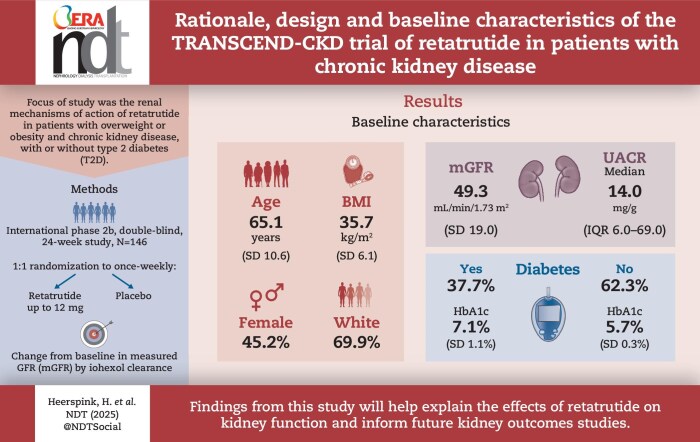

KEY LEARNING POINTS **What was known:**

- Retatrutide is an agonist of the glucose-dependent insulinotropic polypeptide, glucagon-like peptide-1 and glucagon receptors that reduced body weight in participants with obesity and type 2 diabetes (T2D) and may impact key chronic kidney disease (CKD)-related pathophysiological pathways.
- The mechanisms-of-action of retatrutide on kidney function and potential clinical impact are not yet fully understood.

**This study adds:**

- This Phase 2b, 24-week study investigates the kidney-specific mechanisms-of-action of retatrutide in participants with overweight/obesity and CKD, with or without T2D.
- This study integrates gold standard measure of glomerular filtration rate by iohexol clearance, multiparametric kidney magnetic resonance imaging to assess kidney hemodynamics and structure, as well as renal and abdominal adipose tissue, and biomarkers of kidney tissue damage and injury.

**Potential impact:**

- Findings from this study will help explain the effects of retatrutide on kidney function and structure already observed in completed trials and inform ongoing kidney outcomes studies.
- In addition, since retatrutide includes glucagon receptor agonism, the trial is expected to provide mechanistic insights into this pathway in the kidney, where glucagon signaling has been shown to serve an important physiological role.

## INTRODUCTION

Obesity precedes and often accelerates the onset of type 2 diabetes (T2D) and hypertension, both well-known risk factors for chronic kidney disease (CKD) development [1]. Obesity can also cause CKD in individuals without diabetes through mechanisms unrelated to hyperglycemia. In recent years, new therapies have become available for the management of CKD, including sodium-glucose cotransporter-2 inhibitors (SGLT2i) and a non-steroidal mineralocorticoid receptor antagonist. Despite these new therapies, the residual risk remains high and the prevalence of CKD continues to rise and is estimated to affect more than 800 million people globally [2]. Additional therapies to improve the pharmacological management of CKD are urgently needed.

The discovery of the gut hormones glucagon-like peptide-1 (GLP-1) and glucose-dependent insulinotropic polypeptide (GIP) has transformed the management of T2D [3]. In addition, these incretin receptor agonists (RAs) reduce obesity and associated cardiometabolic complications [3]. Specifically, GLP-1 RAs have consistently been shown to confer cardiovascular protection in individuals with T2D and/or obesity [4–6]. In addition, a GLP-1 RA, semaglutide, slowed the progression of CKD and reduced the risks of clinically meaningful kidney and cardiovascular outcomes in patients with T2D and CKD [7]. A dual GIP and GLP-1 RA, tirzepatide, has been approved for the treatment of T2D, obesity and obstructive sleep apnea. Secondary analyses from clinical trials have shown tirzepatide to reduce albuminuria in people with and without T2D compared with placebo or insulin-based treatments [8, 9]. In addition, tirzepatide slowed the decline in estimated glomerular filtration rate (eGFR) compared with insulin glargine in participants with T2D and high cardiovascular disease risk [10].

Emerging insights into the role of glucagon in metabolism and energy expenditure have motivated the development of specific agonists, such as retatrutide, that target the GIP, GLP-1 and glucagon receptors [11]. In experimental models, retatrutide increased energy expenditure and reduced calorie intake [12]. In two Phase 2 clinical trials in participants with T2D and in participants with overweight or obesity without T2D, retatrutide improved glycemic control and reduced body weight along with reductions in blood pressure, cholesterol and markers of inflammation [13, 14]. A dedicated kidney analysis from these two clinical trials focusing on changes in eGFR and albuminuria over time, reported an initial decrease in eGFR with retatrutide, followed by an eGFR increase to baseline values in patients with T2D and even exceeding baseline eGFR in participants with overweight or obesity and without T2D [15]. The eGFR increase at 24 weeks with retatrutide in those with obesity or overweight could reflect an increase in glomerular pressure, potentially leading to single nephron hyperfiltration and structural damage. In earlier studies in both rodents and humans, glucagon infusion was shown to increase GFR, likely by reducing pre-glomerular arteriolar resistance [16, 17]. However, such hemodynamic changes would typically increase albuminuria, which instead decreased significantly with retatrutide versus placebo or dulaglutide, particularly in those with more severe albuminuria [15]. It is also possible that a decrease in body surface area due to weight loss contributes to the increase in eGFR when indexed for 1.73 m^2^ body surface area [18]. The mechanisms contributing to the increase in eGFR are unclear. We hypothesize that retatrutide reduces peri-renal and intra-abdominal adipose tissue, thereby relieving glomerular compression and improving kidney hemodynamic function. It is also possible that reductions in body composition and fat mass during retatrutide treatment affect endogenous filtration markers such as creatinine or cystatin C without real effects on glomerular filtration. A dedicated mechanism-of-action trial on kidney function and structure is therefore critical to elucidate the mechanism of retatrutide on GFR and albuminuria and to interpret and contextualize the forthcoming clinical findings from the Phase 3 TRIUMPH-Outcomes trial (NCT06383390) investigating the effects of retatrutide on cardiovascular and kidney outcomes in participants with obesity, with estimated completion in 2029. We here describe the study design and baseline characteristics of the TRANSCEND-CKD randomized, placebo-controlled trial designed to characterize the mechanism of action of retatrutide on kidney function and structure.

## MATERIALS AND METHODS

### Study design and participants

**Figure 1:**
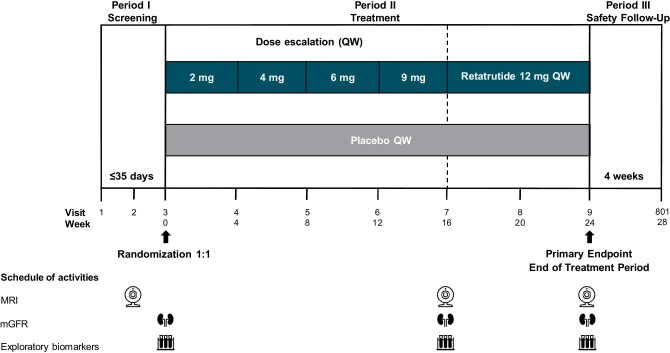
Study design of the Phase 2b renal mechanism-of-action study of retatrutide. QW, once weekly.

This is a Phase 2b, double-blind, 24-week study (NCT05936151) conducted at 42 sites in Canada, Italy, Spain, the UK and USA. The study included a screening period, a treatment period and a safety follow-up period (Fig. 1). Participants were randomly assigned in a 1:1 ratio to once-weekly subcutaneous injection of retatrutide maximum tolerated dose up to 12 mg or matching placebo. Randomization was stratified by T2D status at baseline (with or without T2D), eGFR category (≥25 to <45, ≥45 to <60 and ≥60 to ≤75 mL/min/1.73 m^2^) based on cystatin C eGFR, and treatment with SGLT2i at baseline (yes or no). Retatrutide was initiated at 2 mg once weekly, and the dose was increased every 4 weeks until the randomization dose of 12 mg was achieved (Fig. 1). If the 12 mg dose was not well tolerated, the 4, 6 or 9 mg dose was allowed. All the participants received a lifestyle intervention that included regular individualized counselling sessions for a healthy diet delivered by a dietitian or qualified health care professional. An increased physical activity to at least 150 min/week was also encouraged.

Eligible participants were adults with overweight or obesity [body mass index (BMI) ≥27 kg/m^2^] and established CKD at screening, with or without T2D, on stable treatment for at least 90 days before screening with an angiotensin-converting enzyme (ACE) inhibitor or angiotensin II receptor blockers (ARB) that was considered the maximal appropriate dose per local label and by the investigator for treatment of CKD or hypertension (Table 1). The use of SGLT2i was also allowed if stable for at least 90 days before screening.

**Table 1:** Key inclusion and exclusion criteria of the Phase 2b renal mechanism-of-action study of retatrutide.

Inclusion criteria	Exclusion criteria
• 18 years of age or older at the time of signing the informed consent	• Self-reported change in body weight >5 kg (11 lbs) within 90 days before screening
• BMI ≥27 kg/m^2^• Eithero No T2D diagnosis with an HbA_1c_ <6.5% (<48 mmol/mol) Oro T2D diagnosis with an HbA_1c_ ≤9.5% (≤80.34 mmol/mol) and treated with diet and exercise only or with stable doses of up to three oral antihyperglycemic medications per local labeling, such as metformin, sulfonylurea, meglitinides, SGLT2i or thiazolidinediones, with or without basal insulin, for at least 90 days before screening• CKD diagnosis and eGFR ≥25 to ≤75 mL/min/1.73 m^2^, calculated using cystatin C–based CKD-EPI equation• On stable treatment, for at least 90 days before screening, with an ACE inhibitor or ARB that was considered the maximal appropriate dose per local label and by the investigator for treatment of CKD or hypertension. The use of SGLT2i was allowed in participants without T2D, in which case, the dose was stable for at least 90 days before screening^a^	• Used in 90 days before screening any of the following antihyperglycemic medications: DPP4 inhibitors, amylin analogs, GLP-1 RAs, GIP/GLP-1 RA and short-acting or rapid-acting insulins or U500 Insulin• Prior or planned surgical treatment for obesity• Type 1 diabetes• Acute or chronic hepatitis• History of malignant disease within 5 years before screening

^a^Participants who were intolerant to ACE inhibitors or ARBs were allowed to enter the study.

DPP4: Dipeptidyl Peptidase IV.

### Ethics

TRANSCEND-CKD is being conducted in accordance with ethical principles derived from the Declaration of Helsinki, the Council for International Organizations of Medical Sciences International Ethical Guidelines, the International Council for Harmonisation Good Clinical Practice Guidelines and all applicable laws and regulations. Before the study was initiated, the protocol, protocol amendments, informed consent and other forms were reviewed and approved by a local independent review board/ethics committee. All study participants provided written, informed consent before study-specific procedure commenced. All participants were informed that their participation in the study was voluntary, and they may withdraw their consent at any time.

### Objectives and endpoints

The primary objective of this study was to compare the effect of retatrutide versus placebo for the change in measured glomerular filtration rate (mGFR) by iohexol clearance from baseline to Week 24 (Table 2). Secondary objectives included comparing the effect of retatrutide versus placebo for the change in mGFR from baseline to Week 16 and comparing the effect of retatrutide versus placebo for the change in glomerular and tubular functional measurements, magnetic resonance imaging (MRI)-assessed kidney hemodynamic function and volumetric measurements and kidney injury and function, kidney oxygenation measured by kidney blood oxygenation level-dependent (BOLD) MRI, and body weight from baseline to Week 16 and from baseline to Week 24 (Table 2). Exploratory objectives included comparing the effect of retatrutide to placebo for the change from baseline to Week 16 and from baseline to Week 24 in kidney and abdominal adipose tissue, and renal diffusion, assessed using non-invasive, multimodal MRI techniques, and creatinine- and cystatin C–based eGFR. Adverse events and serious adverse events were included in the safety assessments.

**Table 2:** Primary and secondary objectives and endpoints of the Phase 2b renal mechanism-of-action study of retatrutide.

Objectives	Endpoints
**Primary**	
To compare the effect of retatrutide vs placebo on mGFR from baseline to Week 24	Change in mGFR (mL/min/1.73 m^2^) using iohexol clearance
**Secondary**	
To compare the effect of retatrutide vs placebo on mGFR from baseline to Week 16	Change in mGFR (mL/min/1.73 m^2^) using iohexol clearance
To compare the effect of retatrutide vs placebo from baseline to Week 16 and baseline to Week 24 for	
• Glomerular and tubular functional measurements	• Change in:
	o UACR^a^
	o Creatinine-corrected FENa
	o Filtration fraction estimated from mGFR and MRI-assessed renal mean arterial flow, corrected by hematocrit
	o Markers of glomerular and tubular function including nephrin KIM-1, NAG, LFABP, clusterin and IL-18
• Renal hemodynamic measurements by MRI	• Change in:
	o Mean arterial flow
	o Renal artery resistive index
	o Renal blood flow velocity (PSV and EDV)
	o Global renal perfusion
• Renal volumetric measurements assessed by MRI	• Change in:
	o Total renal parenchyma volume
	o Renal cortex volume
• Renal injury and function by MRI	• Change in:
	o Renal cortex T_1_ (ms)
	o Renal medulla T_1_ (ms)
• Kidney oxygen availability measured by kidney BOLD MRI	• Change in:
	o Renal cortex R_2_*
	o Renal medulla R_2_*
• Body weight	• Percent change in body weight
To compare the effect of retatrutide vs placebo on glomerular and tubular functional measurements from baseline to Week 24	• Change in 24-h UAE (mg/24 h)
	• Change in 24-h urinary electrolytes (mg/24 h)

a Change in UACR will be analyzed in a subset of participants with baseline UACR ≥30 mg/g.

BOLD, blood oxygenation level-dependent; EDV, end-diastolic velocity; FENa, fractional urinary sodium excretion; IL, interleukin; KIM-1, kidney injury molecule 1; PSV, peak systolic velocity; R_2_*, the inverse of transverse relaxation time constant measured by MRI; T_1_, longitudinal relaxation time of tissue measured by MRI; UAE, urinary albumin excretion.

### Assessments and laboratory measurements

#### Iohexol-measured GFR and estimated GFR

The iohexol clearance procedure in this study has been modified for individuals with obesity and CKD. In short, 5 mL of iohexol (Omnipaque 300, 647 mg/mL), equivalent to 3235 mg iohexol, will be slowly injected over 2 min through an intravenous cannula. The exact dose of iohexol will be recorded by weighing the syringe before and after injection. Blood samples for iohexol measurements will be drawn at 120, 150, 180, 210 and 240 min through a separate cannula. Blood samples will be processed according to the central laboratory-provided standard operating procedures, and shipped to the central laboratory for measurement of the iohexol concentration, using liquid chromatography mass spectrometry, and GFR assessment. The plasma iohexol concentration disappearance slope will be visualized for quality control purposes. Obvious outlier concentration (implausible results) will be excluded for a more accurate mGFR calculation as described in the consensus statement of the European Kidney Function Consortium on the iohexol plasma clearance measurement standardization [19]. Creatinine and cystatin C will also be measured at baseline, Weeks 4, 12, 16 and 24, as well as after a 4-week wash-out (Week 28) to estimate GFR using the Chronic Kidney Disease Epidemiology Collaboration (CKD-EPI) 2021 equation [20]. Analyses of measured and estimated GFR will be performed with and without indexation for body surface area.

#### Multiparametric MRI

Multiparametric MRI will be performed at baseline, Weeks 16 and 24 to assess effects of retatrutide on renal hemodynamic, microstructural, volumetric (fat contents and kidney volume) and kidney oxygen availability (Table 2). All MRI sequences will be sent to an external imaging vendor for centralized analysis. If the MRI scan is deemed to be of insufficient quality to obtain adequate measures for the study of secondary objectives, a repeat scan can be scheduled to optimize data quality. The time course of absolute and percentage change from baseline in MRI markers, as described in Table 2, will be summarized in the retatrutide and placebo groups.

#### Urinary albumin excretion

Spot urine samples will be collected at baseline, Weeks 4, 12, 16 and 24, and after a 4-week wash-out (Week 28) for assessment of urine albumin–creatinine ratio (UACR). Twenty-four-hour urine samples are also collected at baseline and Week 24 to determine 24-h albumin excretion rate.

#### Glomerular and tubular functional measurements

Blood and urine samples are collected at baseline, and Weeks 16 and 24 to determine markers reflecting glomerular and tubular function including nephrin, urinary kidney injury molecule 1, N-acetyl-β-glucosaminidase (NAG), liver fatty acid binding protein (LFABP), clusterin and interleukin-18. These markers will be measured in a central laboratory using well-established commercially available assays.

#### Mechanistic biomarkers

Biomarker analysis in stored blood and urine samples will be performed to examine the mechanism of action of retatrutide in participants with CKD. Specifically, to determine changes in insulin sensitivity and effects on the renin–angiotensin–aldosterone system (RAAS), insulin, C-peptide, plasma glucose, plasma renin activity, angiotensinogen and aldosterone will be measured. To study the effects of retatrutide on regulation of calcium and phosphate, parathyroid hormone, 25-OH vitamin D, calcium and phosphate will be measured. Effects of retatrutide on markers of cardiovascular risk including ApoB, APoC3 and N-terminal pro-B-type natriuretic peptide (NT-proBNP) will also be measured. Co-peptin, a stable precursor of anti-diuretic hormone, cyclic adenosine monophosphate (cAMP) and glucagon will be measured to study effects of retatrutide on the glucagon–vasopressin–urea pathway. Moreover, urinary metabolomics will be performed using the Metabolon platform to assess glucagon-mediated effects on amino acid metabolism and changes in renal carbohydrate, lipid, protein and mitochondrial metabolism. In addition, serum large-scale proteomic analysis will be performed to assess changes in protein metabolism.

### Statistical considerations

#### Sample size determination

A sample size of 120 randomized participants, approximately 60 participants in each of the retatrutide and placebo arms, was calculated to provide at least 80% power to establish a significant difference in the change from baseline to Week 24 in mGFR between retatrutide and placebo. The calculation assumed a 15% dropout rate, a two-sided two-sample *t*-test with significance level of 0.05, a standard deviation (SD) of 8.1 and a mean change from baseline in mGFR of 4.56 mL/min/1.73 m^2^ at Week 24 in participants receiving retatrutide compared with placebo based on eGFR data from the Phase 2 studies [13–15].

#### Statistical analyses

The main analysis includes participants who were randomly assigned a study intervention and took at least one dose of study intervention and used the efficacy estimand. The efficacy estimand is based on the hypothetical strategy to address intercurrent events [21]. In this hypothetical strategy, the primary clinical question of interest asks what the treatment difference is in mGFR change from baseline at 24 weeks between retatrutide and placebo in participants who meet the eligibility criteria if they would remain on their randomly assigned treatment for 24 weeks and would not initiate new SGLT2i, ACE inhibitor or ARB. In the efficacy estimand, a mixed model repeated measures analysis model will be used to analyze continuous measurements with multiple postbaseline measures with terms of treatment, visit, stratification factors, baseline measurement, treatment-by-visit interaction and baseline measurement-by-visit interaction. An analysis of covariance will be used to analyze continuous measurements with only one post-baseline assessment with terms of treatment, stratification factors and baseline measurement. The missing data will be imputed using non-missing data within the same treatment group at the same visit under the missing at random assumption. The estimated treatment difference between retatrutide and placebo will be presented along with the two-sided 95% confidence interval and *P*-value. No adjustments for multiplicity will be performed for primary and secondary objectives. Safety assessments used an estimand comparing the safety of retatrutide with that of placebo, irrespective of adherence to study intervention.

## RESULTS

### Setting and participant disposition

Participants were recruited between 20 July 2023 and 28 February 2025. A total of 367 participants were screened for eligibility, of whom 146 were randomized to study intervention.

### Baseline characteristics of participants

The mean age of the overall population was 65.1 years (SD 10.6), 45.2% were female and 69.9% were White (Table 3). Participants had a mean baseline body weight of 101.1 kg (SD 20.6), waist circumference of 115.5 cm (SD 14.3) and BMI of 35.7 kg/m^2^ (SD 6.1). T2D was diagnosed in 37.7% of participants, with a mean baseline hemoglobin A_1c_ (HbA_1c_) of 7.1% (SD 1.1%), while the baseline HbA_1c_ in participants without T2D was 5.7% (SD 0.3%).

**Table 3:** Baseline demographics and clinical characteristics of the randomized participants in the Phase 2b renal mechanism-of-action study of retatrutide.

Variable	Overall population (*N* = 146)
Age, years (SD)	65.1 (10.6)
Sex, *N* (%)	
Female	66 (45.2)
Male	80 (54.8)
Race, *N* (%)	
American Indian or Alaska Native	1 (0.7)
Asian	15 (10.3)
Black or African American	24 (16.4)
White	102 (69.9)
Not reported	4 (2.7)
Ethnicity, *N* (%)	
Hispanic or Latino	19 (13.0)
Not Hispanic or Latino	122 (83.6)
Not reported	5 (3.4)
Waist circumference, cm (SD)	115.5 (14.3)
Body weight, kg (SD)	101.1 (20.6)
BMI, kg/m^2^ (SD)	35.7 (6.1)
Tobacco use, *N* (%)	
Yes	69 (47.3)
No	77 (52.7)
Diabetes status, *N* (%)	
Yes	55 (37.7)
No	91 (62.3)
Duration of T2D for participants with T2D diagnosis, years (SD)	10.0 (8.1)
HbA_1c_ for participants with T2D, % (SD)	7.1 (1.1)
HbA_1c_ for participants without T2D, % (SD)	5.7 (0.3)
Concomitant therapy, *N* (%)	
Antihypertensive agents	134 (94.4)
ACE inhibitors	51 (35.9)
ARB	83 (58.5)
Diuretics	3 (2.1)
Lipid-lowering agents	106 (74.6)
Antihyperglycemic agents	53 (37.3)
Metformin	36 (25.4)
SGLT2i	31 (21.8)
Sulfonylurea	6 (4.2)
Basal insulin	6 (4.2)
DPP4 inhibitors	1 (0.7)
Systolic blood pressure, mmHg (SD)	130.5 (16.4)
Diastolic blood pressure, mmHg (SD)	78.2 (9.6)
BSA indexed mGFR mL/min/1.73 m^2^ (SD)	49.3 (19.0)
Cystatin C–based eGFR, mL/min/1.73 m^2^ (SD)	49.6 (13.2)
Creatinine-based eGFR, mL/min/1.73 m^2^ (SD)	64.2 (17.8)
eGFR category, *N* (%)	
≥25 to <45 mL/min/1.73 m^2^	53 (36.3)
≥45 to <60 mL/min/1.73 m^2^	58 (39.7)
≥60 to ≤75 mL/min/1.73 m^2^	35 (24.0)
UACR, mg/g, median (IQR)	14.0 (6.0–69.0)
UACR category, *N* (%)	
<30 mg/g	102 (69.9)
≥30 mg/g	44 (30.1)
Triglycerides, mg/dL (SD)	143.4 (84.4)
Total cholesterol, mg/dL (SD)	162.5 (42.9)
LDL-cholesterol, mg/dL (SD)	86.0 (35.3)
HDL-cholesterol, mg/dL (SD)	48.0 (15.6)
Previous CVD (myocardial infarction or stroke), *N* (%)	15 (10.3)
Heart failure, *N* (%)	7 (4.8)

Data are reported as means with SD, medians with interquartile range (IQR) or numbers with percentage (%).

BSA, body surface area; CVD, cardiovascular disease; DPP4, Dipeptidyl Peptidase IV; HDL, high-density lipoprotein; LDL, low-density lipoprotein.

The mean baseline iohexol mGFR was 49.3 mL/min/1.73 m^2^ (SD 19.0), the cystatin C–based eGFR was 49.6 mL/min/1.73 m^2^ (SD 13.2) and the creatinine-based eGFR was 64.2 mL/min/1.73 m^2^ (SD 17.8). The median baseline UACR was 14.0 mg/g (interquartile range 6.0–69.0), with 30.1% of participants having ≥30 mg/g at baseline.

A total of 35.9% of participants were receiving ACE inhibitors, and 58.5% were on ARB. Overall, 74.6% of participants were taking lipid-lowering medications, 21.8% were using SGLT2i, 25.4% metformin, 4.2% sulfonylurea and 4.2% basal insulin. Other baseline clinical characteristics are presented in Table 3.

## DISCUSSION

The TRANSCEND-CKD trial was designed to characterize the effects of retatrutide on kidney function and structure in participants with CKD with and without T2D. Based on Phase 2 studies demonstrating pronounced improvements in metabolic parameters, including body weight, blood pressure, HbA_1c_ and cholesterol, as well as reductions in albuminuria [13–15], there is a sound rationale that retatrutide will provide long-term kidney protection. The observed increase in eGFR after 24 weeks of treatment with retatrutide in patients with overweight or obesity, but not in those with T2D [15], required a dedicated mechanism-of-action study to elucidate the underlying kidney-related pharmacological effects of retatrutide.

Although retatrutide reduced traditional risk factors for CKD progression, such as HbA_1c_, blood pressure and body weight, it may also confer kidney protection by directly activating the GIP, GLP-1 and glucagon receptors. Experimental and clinical studies have shown that GLP-1 receptor–mediated kidney protection involves suppression of inflammation and oxidative stress [22–24]. In addition, in healthy volunteers, GLP-1 receptor activation promoted kidney oxygenation through reducing cortical and medullary perfusion and increasing natriuresis, which may reduce oxygen consumption by reducing tubular sodium reabsorption [25]. The direct effects of GIP receptor activation on the kidney are not well established, most likely because the expression of GIP receptors is very low in kidney tissue. However, GIP is expressed in white adipose tissue where it is involved in insulin sensitization, glucose uptake, triglyceride storage and lipolysis [26]. Targeting GIP in ectopic renal fat might attenuate adipose tissue inflammation and, in turn, improve kidney function [27].

Activation of the glucagon receptor by retatrutide is expected to influence several kidney processes. While glucagon traditionally was considered as a glucoregulatory hormone that maintains euglycemia during hypoglycemic challenges by increasing hepatic glucose production, it also plays a key role in kidney physiology [28]. Together with vasopressin, glucagon facilitates excretion of protein waste products, including urea. In the distal tubule, glucagon receptor activation via cAMP signaling promotes sodium, potassium and phosphate excretion [29]. Experimental studies suggest that glucagon–vasopressin–urea interactions reduce tubuloglomerular feedback, potentially increasing renal blood flow and intraglomerular pressure, leading to single nephron hyperfiltration and elevated whole-kidney GFR [30]. However, clinical data supporting this hypothesis remain lacking. The TRANSCEND-CKD trial will measure co-peptin, cAMP and glucagon before and after retatrutide treatment to explore whether these experimental findings translate to humans. In addition, by using iohexol and MRI-based perfusion data, measures of intra-kidney hemodynamic function will be assessed.

In addition to its glycocentric effects, glucagon also increases energy expenditure and reduces food intake through various postulated mechanisms, inducing weight reduction [31]. When combined with GLP-1 and GIP receptor activation, glucagon agonism is expected to cause a marked reduction in perirenal adipose tissue. A reduction in perirenal adipose tissue and possibly renal sinus fat may have a direct mechanical impact on the kidney, alleviating compression of renal parenchyma and blood vessels, as previously observed in experimental rabbit and dog studies [32–34]. However, human mechanistic data to support this hypothesis is lacking. In addition, changes in adipose tissue biology by retatrutide could change the composition of the adipose tissue secretome and thereby directly modulate vascular function and kidney perfusion [35]. These effects may increase renal blood flow and GFR in the absence of an increase in glomerular pressure or hyperfiltration and may explain the previously observed increase in GFR with retatrutide in adults with overweight or obesity [15]. It is also likely that retatrutide reduces the RAAS, mediated by a reduction in adipocytes, which synthesize RAAS components. Along with reductions in insulin resistance and renal sympathetic nervous system, this may increase natriuresis, decrease systemic blood pressure and have beneficial intraglomerular hemodynamic consequences. The pre-specified biomarker assessments in the TRANSCEND-CKD trial will provide more insight into the effects of retatrutide on these mechanistic pathways. In addition, the trial employs MRI to study the effects of retatrutide on subcutaneous, visceral, renal sinus and perirenal adipose tissue volumes. MRI also enables assessment of mean renal arterial blood flow. When renal blood flow is corrected for hematocrit, renal plasma flow and filtration fraction can be calculated, which will help elucidate whether the observed increase in mGFR may be attributed to increased glomerular pressure and hyperfiltration or can be attributed to other mechanisms secondary to weight loss.

Recent work reframes the kidney as both a glucagon target and a major clearance organ [36]. Glucagon receptor is localized across nephron segments, enriched in thick ascending limb/distal tubule, and its expression is reduced in human CKD and correlates with eGFR [37, 38]. Mechanistically, kidney-specific glucagon receptor deletion in mice induced hyperaminoacidemia, reduced renal glucose output, oxidative stress, inflammasome activation, lipotoxicity and progressive fibrosis, establishing a homeostatic role for the glucagon receptor in the kidney [37]. Conversely, sustained receptor activation (modeling chronic hyperglucagonemia) drives mesangial expansion and albuminuria, with transcriptomic shifts in fatty acid metabolism and Na^+^, K^+^ ATPase pathways, implying that excess signaling is maladaptive [39].

GFR estimation equations may be affected by significant weight loss, as changes in creatinine or cystatin C may reflect reduction in muscle or fat mass without actual changes in glomerular filtration. In a previous Phase 2 study in people with T2D, no correlations were observed between changes in creatinine- or cystatin C–derived eGFR and changes in body weight, suggesting that changes in body weight did not impact eGFR [15]. However, in adults with overweight or obesity, a change in body weight after 48 weeks of treatment with retatrutide at a dose of 12 mg once weekly correlated with an increase in creatinine-based eGFR [15]. This was not observed for cystatin C–based eGFR, suggesting that cystatin C–based eGFR is the preferred method to monitor eGFR during treatment with retatrutide. However, the prior studies did not measure GFR and included only a limited number of patients with CKD. Comparing changes in mGFR with eGFR by creatinine or cystatin C during retatrutide treatment in the TRANSCEND-CKD will inform the optimal method to monitor changes in kidney function over time. To characterize acute changes in mGFR upon initiation of retatrutide, an additional GFR measurement at 4 weeks was considered during the design of the study, but decided not to include due to feasibility and operational concerns. The collection of 24-h urine samples in TRANSCEND-CKD also allows comparison of 24-h creatinine clearance with other GFR assessment. Changes in lean body weight may also affect 24-h creatinine clearance and impact effects of retatrutide on UACR, as previously noted [40]. The effects of retatrutide on 24-h urinary albumin excretion will be assessed in the current study to determine whether changes in urinary creatinine excretion underestimate effects on UACR.

Other mechanistic studies using measured and estimated GFR with incretin-based therapies are completed or ongoing (Table 4). The SMART study (SeMaglutide and Albuminuria Reduction Trial in Obese Individuals Without Diabetes) assessed the effects of semaglutide in adults with CKD and overweight or obesity but without diabetes by measuring GFR by iohexol clearance, as well as creatinine- or cystatin C–based eGFR. Similar to our study, baseline creatinine-based eGFR was higher compared with mGFR, which may reflect a non-GFR-related effect on serum creatinine [41]. The SMART study showed that semaglutide compared with placebo reduced body weight over 24 weeks of treatment by 9.1 kg but did not change mGFR or eGFR [41]. The study reported no correlations between changes in body weight and changes in either mGFR or eGFR during semaglutide treatment. Semaglutide is also being studied in adults with CKD and T2D [42]. This study has completed the recruitment of 106 participants. Although the study did not measure GFR, it included multiparametric MRI, creatinine clearance from 24-h urine collections, and paired kidney biopsies with advanced molecular profiling were performed in a subgroup of 33 participants [42]. These data will provide in-depth insight into the mechanisms of action of semaglutide on kidney function in people with T2D and CKD. Another ongoing study (TREASURE-CKD) assesses the effects of 52 weeks of treatment with the GIP/GLP-1 RA tirzepatide on mGFR and eGFR, as well as multi-modal imaging integrating MRI and positron emission tomography (PET) technologies to characterize kidney metabolism. This study aims to enroll 140 patients with and without T2D (NCT05536804). In contrast to these studies, TRANSCEND-CKD did not include a UACR threshold for inclusion as the change from baseline in mGFR is the primary endpoint. Enrolling a broad population with varying degrees of albuminuria allows us to characterize the change in mGFR in participants with no (KDIGO A1), moderate (KDIGO A2) and severe albuminuria (KDIGO A3).

**Table 4:** Comparison of mechanistic studies with incretin-based therapies in participants with CKD.

	SMART (NCT04889183)	REMODEL (NCT04865770)	TREASURE-CKD (NCT05536804)	TRANSCEND-CKD (NCT05936151)
Study population	Overweight/obesity without T2D	T2D	Overweight/obesity with and without T2D	Overweight/obesity with and without T2D
eGFR/UACR inclusion criteria	≥25 mL/min/1.73 m²	≥30 to ≤75 mL/min/1.73 m²	≥25 to ≤60 mL/min/1.73 m²	≥25 to ≤75 mL/min/1.73 m²
	and	and	or	
	UACR ≥30 and <3500 mg/g	UACR ≥20 and <5000 mg/g	≥25 to ≤75 mL/min/1.73 m² if UACR >30 mg/g	
Incretin	Semaglutide	Semaglutide	Tirzepatide	Retatrutide
Design	Randomized placebo-controlled clinical trial	Randomized placebo-controlled clinical trial	Randomized placebo-controlled clinical trial	Randomized placebo-controlled clinical trial
Treatment period (weeks)	24	52	52	24
Sample size	101	106	140^a^	146
Primary Outcome	Change from baseline in UACR	MRI-based outcomes, including change in kidney oxygenation and perfusion	Change from baseline in kidney oxygenation	Change from baseline in mGFR
Assessments	mGFR	Multiparametric MRI	mGFR	mGFR
	Bioimpedance spectroscopy	Kidney tissue (*N* = 33)	PET scans	Multiparametric MRI
	Blood/urine biomarkers	Blood/urine biomarkers	Multiparametric MRI	Blood/urine biomarkers

^a^Planned sample size.

PET, positron emission tomography.

TRANSCEND-CKD is a randomized, placebo-controlled clinical trial that assesses the effects of retatrutide on kidney function and structure. The trial findings will inform future studies of retatrutide and may help contextualize clinical findings in the ongoing cardio-kidney outcome trial TRIUMPH-Outcomes (NCT06383390).

## Data Availability

Lilly provides access to all individual participant data collected during the trial, after anonymization, with the exception of pharmacokinetic or genetic data. Data are available to request 6 months after the indication studied has been approved in the USA and EU and after primary publication acceptance, whichever is later. No expiration date of data requests is currently set once data are made available. Access is provided after a proposal has been approved by an independent review committee identified for this purpose and after receipt of a signed data sharing agreement. Data and documents, including the study protocol, statistical analysis plan, clinical study report, blank or annotated case report forms, will be provided in a secure data sharing environment. For details on submitting a request, see the instructions provided at www.vivli.org
